# Deep-Brain Electrical Microstimulation Is an Effective Tool to Explore Functional Characteristics of Somatosensory Neurons in the Rat Brain

**DOI:** 10.1371/journal.pone.0117289

**Published:** 2015-02-19

**Authors:** Han-Jia Jiang, Kuang-Hsuan Chen, Fu-Shan Jaw

**Affiliations:** Institute of Biomedical Engineering, National Taiwan University, Taipei, Taiwan; University of Salamanca- Institute for Neuroscience of Castille and Leon and Medical School, SPAIN

## Abstract

In neurophysiology researches, peripheral stimulation is used along with recordings of neural activities to study the processing of somatosensory signals in the brain. However, limited precision of peripheral stimulation makes it difficult to activate the neuron with millisecond resolution and study its functional properties in this scale. Also, tissue/receptor damage that could occur in some experiments often limits the amount of responses that can be recorded and hence reduces data reproducibility. To overcome these limitations, electrical microstimulation (ES) of the brain could be used to directly and more precisely evoke neural responses. For this purpose, a deep-brain ES protocol for rat somatosensory relay neurons was developed in this study. Three male Wistar rats were used in the experiment. The ES was applied to the thalamic region responsive to hindpaw tactile stimulation (TS) via a theta glass microelectrode. The resulting ES-evoked cortical responses showed action potentials and thalamocortical relay latencies very similar to those evoked by TS. This result shows that the developed deep-brain ES protocol is an effective tool to bypass peripheral tissue for *in vivo* functional analysis of specific types of somatosensory neurons. This protocol could be readily applied in researches of nociception and other somatosensory systems to allow more extensive exploration of the neural functional networks.

## Introduction

In neurophysiology researches, electrical neural activities measured *in vivo* provide much information about the processing of somatosensory signals (e.g. tactile, nociceptive, etc.) in the brain. Conventionally, adequate stimulus (e.g. mechanical force) is applied to peripheral tissue, and the evoked neural responses are recorded for observation and analyses. However, limitations may exist under certain conditions. Because peripheral stimulus of millisecond precision is not easy to produce or control, it is difficult to activate a somatosensory neuron with such resolution to study its functional properties at this scale. Also, in some experiments such as those of nociception researches, repeated stimulation may cause tissue/receptor damage [1,2]. Once damaged, it becomes difficult to evoke responses from the targeted tissue, and the number of recordings that can be obtained is consequently limited. This often seriously reduces data reproducibility.

Using electrical microstimulation (ES) could overcome these limitations. Because ES is a controllable and precise tool that can be applied to the nervous system without much damage, it has been widely used in researches to probe neural circuitries [3–5], and also in clinical practices to treat neurological disorders and restore sensory functions [6–9]. With proper devices, ES could allow direct stimulation of the neurons with a millisecond resolution or even better. When signal collection is hindered by tissue damage or other problems, it could also be used to bypass the peripheral tissue and repeatedly evoke neural responses. Therefore, more extensive study of the neuron functions in the somatosensory system could be achieved using ES.

For this purpose, a deep-brain ES protocol for the rat thalamus was developed in this study. Considering that tactile stimulation (TS) evokes obvious signals with clear somatotopic organization, and thus allows easier locating of target neurons, the tactile pathway was chosen here to establish the protocol. Using a glass microelectrode, ES was applied to the ventral posterolateral nucleus (VPL) of thalamus to stimulate thalamic relay neurons, the tertiary neurons of the somatosensory system responsible for signal relaying to the cortex. Action potentials (APs) in the primary somatosensory (SI) cortex were successfully evoked by ES, and were compared with those evoked by TS, thereby demonstrating the feasibility of using this protocol to study specific neurons in the brain.

## Materials and Methods

### Animal surgery and ethical statement

Three adult male Wistar rats weighing 250–400 g were used in the experiment. Each animal was kept anesthetized by an initial intraperitoneal injection (50 mg/kg) and subsequent intravenous injections (10 mg/kg, every 1 hour) of sodium pentobarbital. For stimulation and recording of the right hemisphere, a rectangular craniotomy 0–5 mm posterior to bregma and 0.5–5 mm lateral to midline was performed. The body temperature was maintained at 37±0.5°C using a homeothermic blanket. If an increase in breathing frequency (over 80/min) or signs of discomfort (twitching, whisker motion) were observed, an extra dose of pentobarbital (5 mg/kg) was injected intravenously to relieve the animal. At the end of the experiment, the animal was sacrificed by an intravenous injection of pentobarbital overdose (30 mg/kg). All treatments were based on concerns of minimizing animal suffering. The protocol was approved by the Institutional Animal Care and Use Committee (IACUC) of National Taiwan University College of Medicine and College of Public Health (Approval Number: 20120498), valid from August 2013 to July 2014.

### Stimulation and recording

**Fig 1:**
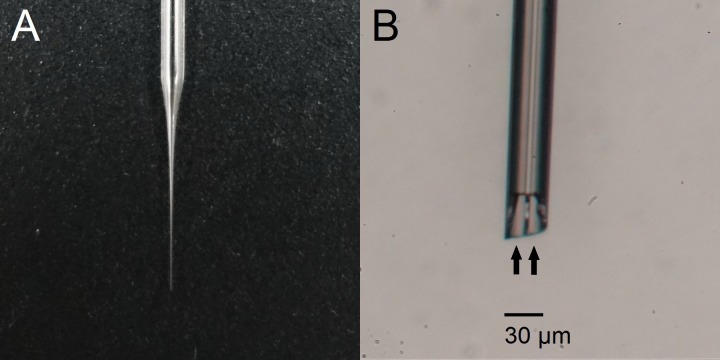
Theta glass electrode. (A) The glass was pulled to produce the tip for microstimulation. (B) The tip of the glass. With two isolated channels (as indicated by the arrows), the tip could be used as a bipolar microelectrode.

Two types of borosilicate glass pipettes were used as electrodes for response searching and subsequent recordings. A normal micropipette (6025, A-M Systems) was used for the cortex, and a theta micropipette (TGC150-10, Warner Instruments) was used for the thalamus. The two micropipettes were processed with a pipette puller (model 720, David Kopf Instruments) to produce tapered tips for insertion. In the ES process, the theta micropipette was used as a bipolar microelectrode (Fig. 1) to deliver electrical pulses, which could prevent extensive spread of charge and thus provide a precision higher than conventional unipolar electrodes [10].

The electrodes were injected with 3 M NaCl solution for conduction and connected to an amplification system that included a self-designed amplifier (×2500), an analog-to-digital card (BNC-2090, National Instruments) for signal conversion, and a computer for storage. Data were collected at a sampling rate of 100 kHz.

The thalamic and cortical neurons responsive to hindpaw tactile stimuli were located by the following method. The electrode tip was inserted into the brain surface and descended progressively in 100 μm steps using a micromanipulator (MO-10, Narishige). Meanwhile, the hindpaw was touched with a brush to evoke response signals, which were turned into sound spikes by an audio monitor (AM8, Grass Technologies) to facilitate aural searching. Once the responses were located, the stimulation and recording processes were started.

The TS was applied to the center of the left hindpaw, and the thalamic and cortical responses were recorded with the two glass electrodes. The two electrodes were then kept in the same position to apply the ES to the thalamus and to record the cortical responses. With this design, the thalamic relay neurons identified by TS could be precisely stimulated by ES, and the TS- and ES-evoked cortical responses could be compared. All recordings were extracellular.

The TS (9 gw) was generated by a self-designed mechanical stimulator and applied with a 0.4 mm diameter nylon stick [11].

The ES was a single pulse generated by a constant-voltage pulse generator (DS2, Digitimer). Multiple levels of parameters were tested in the experiment to evoke cortical responses. These included strengths of 20, 40, 60, 80, and 90 V, and durations of 0.2, 0.6, 1, 1.4, 1.8, and 2.0 ms. With the measured impedance (885 kΩ on average and a minimum of 770 kΩ) of the pulled theta glass tip, the maximum current amplitude was estimated to be 0.12 mA.

### Data analysis

All recordings were digitally low-pass filtered (2nd-order Butterworth filter, at 5 kHz) to reduce environmental high-frequency noise. The following analyses were then conducted for filtered data of each animal. Signal correlations were all evaluated by Pearson’s *r*. All analyses were carried out using MATLAB 7.10.0.

**Fig 2:**
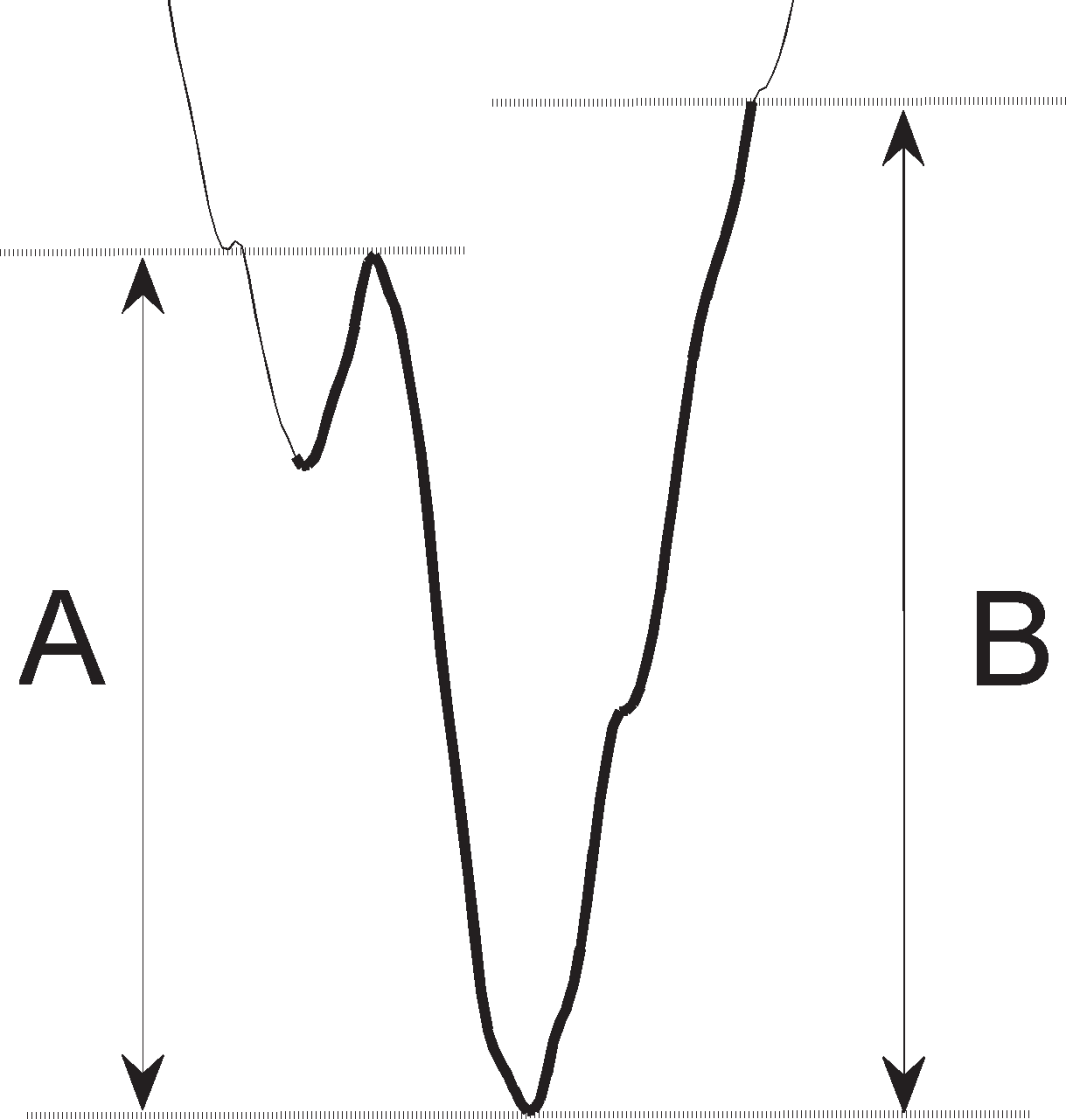
Detection of spike signals. A: descending amplitude. B: ascending amplitude. Signals with both amplitudes larger than the threshold were detected as evoked spikes.

**Spike detection.** For subsequent comparisons, AP spikes in the recorded traces were detected using thresholds. In our data, cortical spikes were generally larger than thalamic spikes, so their detection parameters (thresholds and signal durations) were determined respectively; but the standard remained the same among all subjects. For cortex traces, any 0.7-ms signal with both descending and ascending amplitudes larger than the threshold were detected as TS- or ES-evoked cortical AP spikes (Fig. 2). The adopted threshold was 10 times the size of the baseline activity, which was defined as the standard deviation of the last 10-ms signal (60 to 70 ms) in each trace. The first 5 ms of each trace were excluded from detection because of stimulation artifacts. For thalamus traces, the same method was adopted to detect TS-evoked thalamic AP spikes, but the threshold was 6 times the size of the baseline activity, and a 0.5-ms signal duration was used.

**Fig 3:**
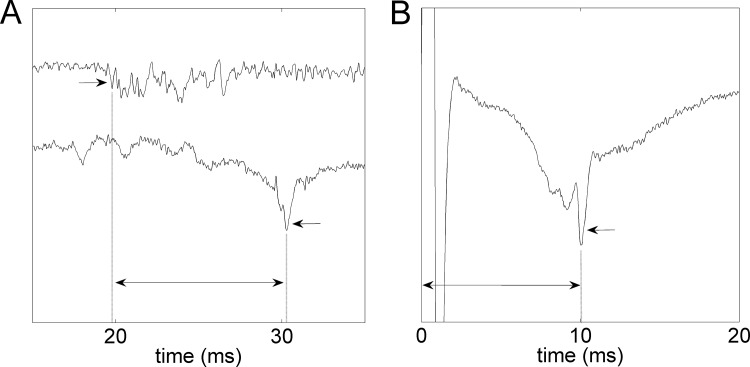
TC-relay latency obtainment. The two traces in (A) are thalamus and cortex traces of the same TS trial. The trace in (B) is the cortex trace of an ES trial. Single arrows indicate detected AP spikes (for the thalamus trace only the first). Double arrows represent the obtained TC-relay latencies.

**Latency comparison.** Thalamocortical relay (TC-relay) latency of each detected cortical spike was obtained. Similarity in this latency would indicate the same relay pathway from thalamus to cortex. For TS, it was defined as the time delay between the cortical spike and the first thalamic spike of the same trial (Fig. 3A). For ES, it was defined as the latency of the cortical spike (Fig. 3B), because ES was applied directly to the thalamus. With the individual latencies thereby obtained, the distribution in each subject was plotted and shown.

**Waveform comparison.** The similarity between TS- and ES-evoked cortical APs in each subject was quantified using averaged signals of selected samples. Firstly, for both TS and ES, 4 spikes in different traces were selected for this purpose, based on their signal correlation. The two spikes with highest correlation among all detected spikes were first selected; then, among the rest, the two that respectively yielded highest correlation with the above two were also selected. Thereby, the selected spikes would share a similar waveform and could thus better represent the evoked response. The correlations were evaluated using the detected 0.7-ms signals.

Subsequently, for both TS and ES, an averaging procedure was performed for the 4 traces with selected spikes, which used time alignment to conserve the spike signals (S1 Fig.): (1) The most highly correlated 0.5-ms segments of 2 selected spikes were identified by examining all 0.5-ms segments with central points within the 0.1-ms centered on the peak. The 2 traces were shifted on the time axis to completely overlap these 2 segments, and then averaged. (2) The other 2 traces were also averaged in the same way. (3) The resulting 2 traces were again averaged in the same way, thus obtaining the trace with the averaged signal of 4 selected spikes.

Finally, the most highly correlated 1-ms segments of the two averaged spike signals of TS and ES were identified by examining all 1-ms segments with central points within the 0.1-ms centered on the peak. The correlation coefficient between these two 1-ms averaged signals was taken as a quantification of the AP waveform similarity in each subject. These signals and the signals in the individual traces that contributed to them were plotted for visual demonstration.

## Results

### Spatial distribution of tactile responses

Among the 3 subjects, the recorded thalamic tactile responses were between 3.30 to 3.80 mm lateral to midline, 2.90 to 3.10 mm posterior to bregma, and 6.30 to 6.65 mm beneath brain surface, while the cortical responses were between 2.40 to 3.00 mm lateral to midline, 1.31 to 1.83 mm posterior to bregma, and 0.74 to 1.23 mm beneath brain surface. These distributions match the VPL nucleus of thalamus and the hind limb regions of SI cortex, according to the atlas provided by Paxinos and Watson [12].

### Comparison of TS- and ES-evoked responses

**Fig 4:**
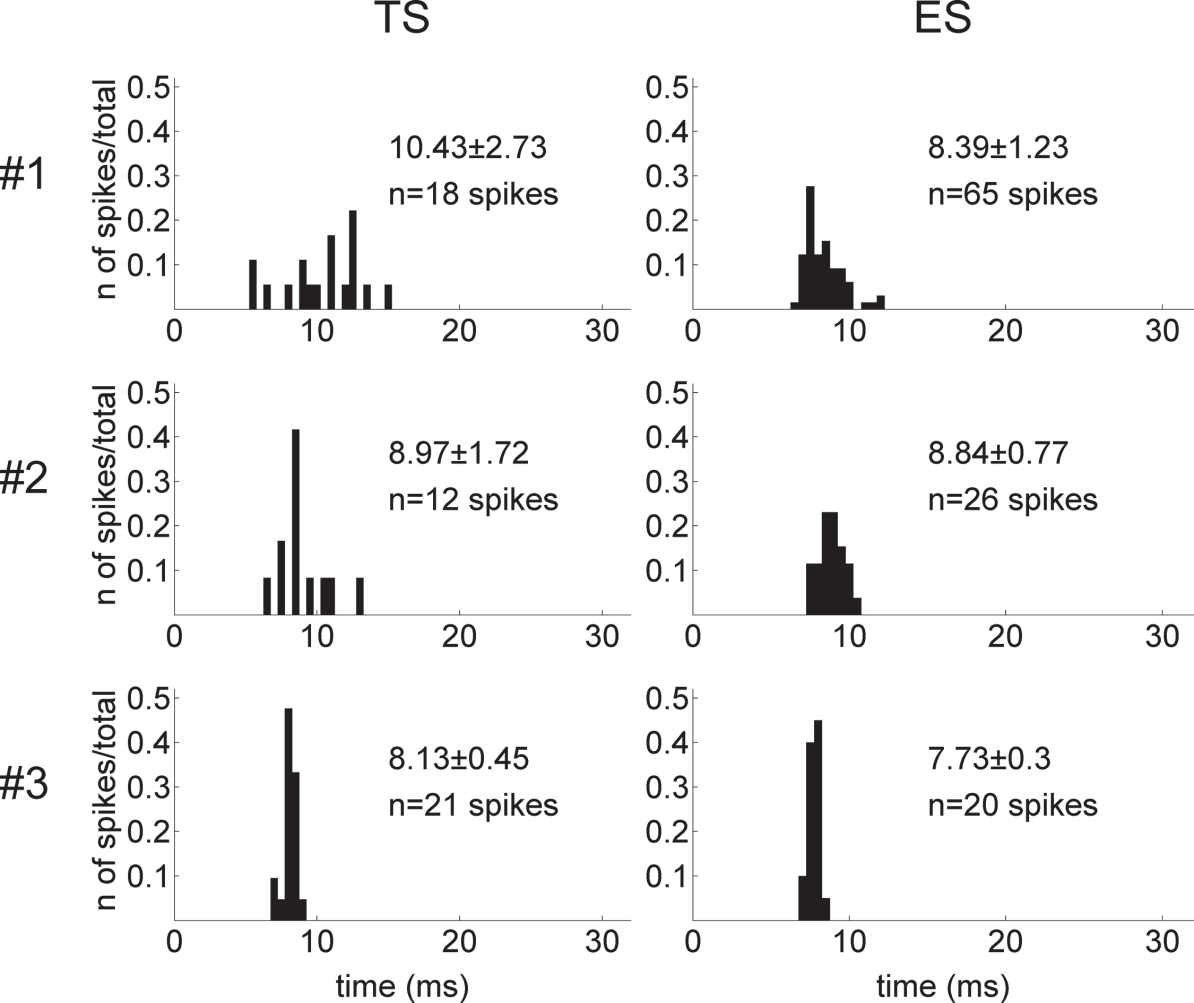
TC-relay latency distribution of detected cortical spikes.

TC-relay latency distributions of the detected cortical AP spikes are shown in Fig. 4. The outliers in each subject (more than 1.5 interquartile ranges below the first quartile or above the third quartile) were excluded. In this result, similar distributions of TS and ES can be seen in each subject. Detected cortical spikes in one subject (#2) are shown in S2 Fig. to demonstrate the reproducibility of the evoked responses.

**Fig 5:**
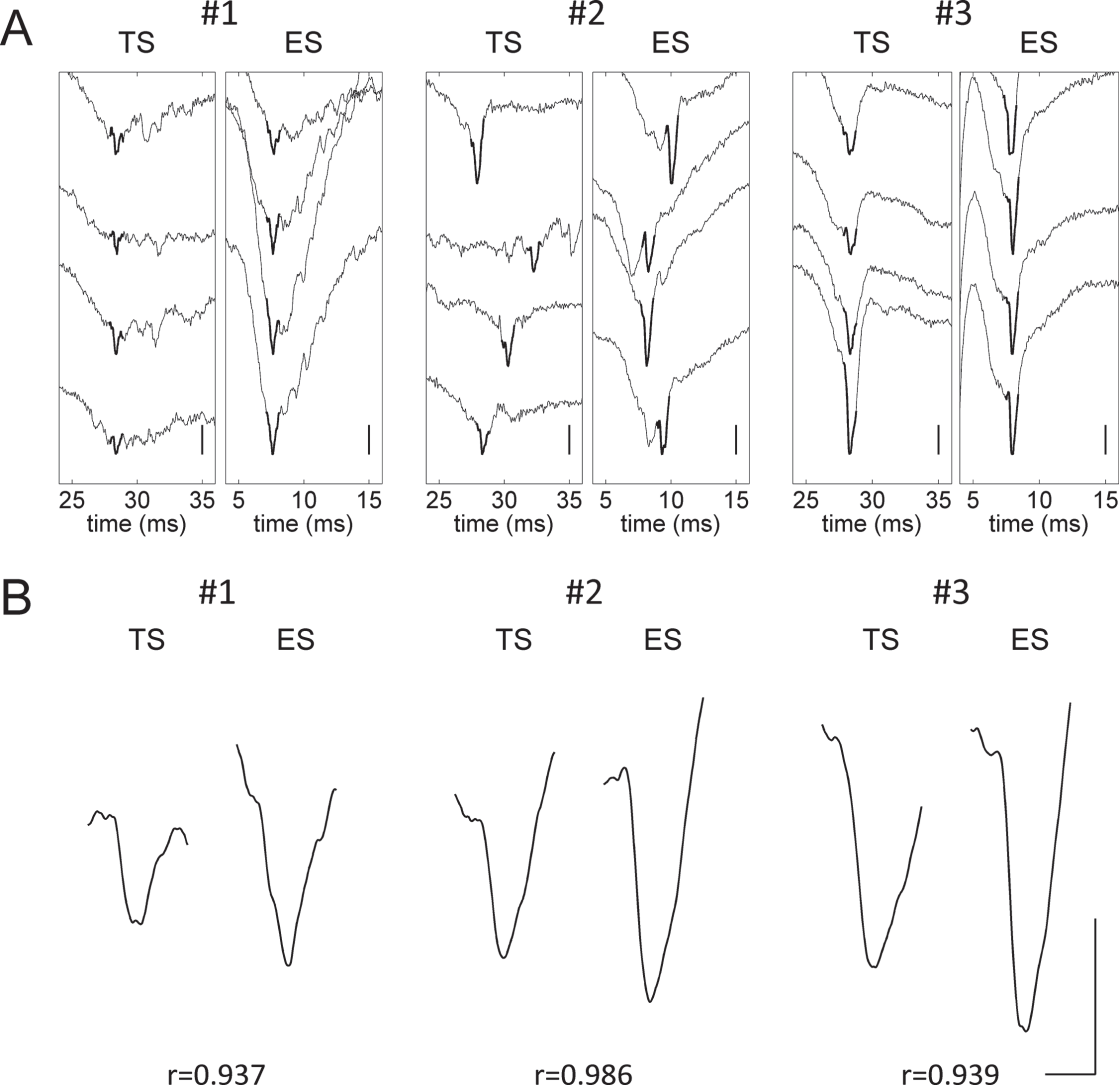
Waveform comparison of TS- and ES-evoked cortical AP responses. (A) The individual traces with selected spikes. Bold lines show the individual 1-ms spike signals contributing to the averaged signals in (B). Scale: 0.2 mV. (B) The 1-ms averaged spike signals and the correlation coefficients yielded by them. Scale: 0.5 ms, 0.2 mV.

A clear similarity between TS- and ES-evoked cortical AP spikes can be seen from the selected samples of each subject (Fig. 5A). In the averaged traces of these samples, 1-ms averaged spike signals (Fig. 5B) that yielded very high correlations (Pearson’s *r*>0.9) between TS and ES were identified. These very high correlations (values in Fig. 5B) support the observed waveform similarity in each subject. The signals in the individual traces that contributed to the averaged signals are shown by bold lines in Fig. 5A.

## Discussion

The detected TS- and ES-evoked responses shared similar TC-relay latency distributions. Also, the high signal correlations yielded by the 1-ms averaged signals of the selected spike samples support the waveform similarity in terms of AP responses. Since the cortex recording electrode was kept in the same position, these results certainly indicate that ES evoked responses of the same cortical neuron (or neuron group) through the same relay pathway as TS did.

In 2 of the 3 subjects, average TC-relay latencies of the detected TS and ES responses were very close to each other, with differences less than 0.5 ms. In the other one (#1), the difference was 2.04 ms (TS>ES). Because this difference was close to the duration of a synaptic delay, we consider that it did not reflect major deviation of the relay pathway. It is possible that the part of the pathway excited by ES was postsynaptic to the signal-generating part.

*In vivo* and direct functional analyses of somatosensory neurons in the thalamus and cortex could be done using this protocol. With the precision of ES, the encoding of thalamic activities in the cortex could be studied with finer resolution. Stimulation strength or duration could be varied to analyze how different degrees of thalamic neuron excitation are reflected in downstream cortical responses. Furthermore, by systematically probing areas of thalamus with ES and observing corresponding cortical responses, more properties of somatotopic and other functional organizations of the thalamocortical projections could be revealed.

We also expect that the problem of tissue damage in nociception experiments could be overcome with this protocol. This application could be carried out by using noxious instead of tactile stimuli to locate nociceptive relay neurons in the thalamus. Once the neurons are located, ES would then be adopted to allow more stimulation cycles. With the larger amount of data that could thereby be obtained, the signals relayed by the nociceptive thalamic neurons to the cortex could be analyzed more extensively than would be possible using noxious stimulation alone. This may substantially contribute to further understanding of the nociceptive system in the brain.

## Supporting Information

S1 Fig Trace averaging procedure. For both TS and ES, the 4 cortex traces with selected spikes were averaged using this procedure. The numbers correspond to the steps described in the text. In each step, two traces were shifted to overlap the most highly correlated signals (bold lines) and then averaged.
(TIF)

S2 Fig Detected cortical spikes in one subject (#2). (A) TS-evoked spikes. (B) ES-evoked spikes. Bold lines show detected signals.
(TIF)
