# Supplementary figures and images for: Deep-Brain Electrical Microstimulation Is an Effective Tool to Explore Functional Characteristics of Somatosensory Neurons in the Rat Brain
Source: PLoS One. 2015 Feb 19;10(2):e0117289. doi: 10.1371/journal.pone.0117289 (PMC4335035; doi:10.1371/journal.pone.0117289)

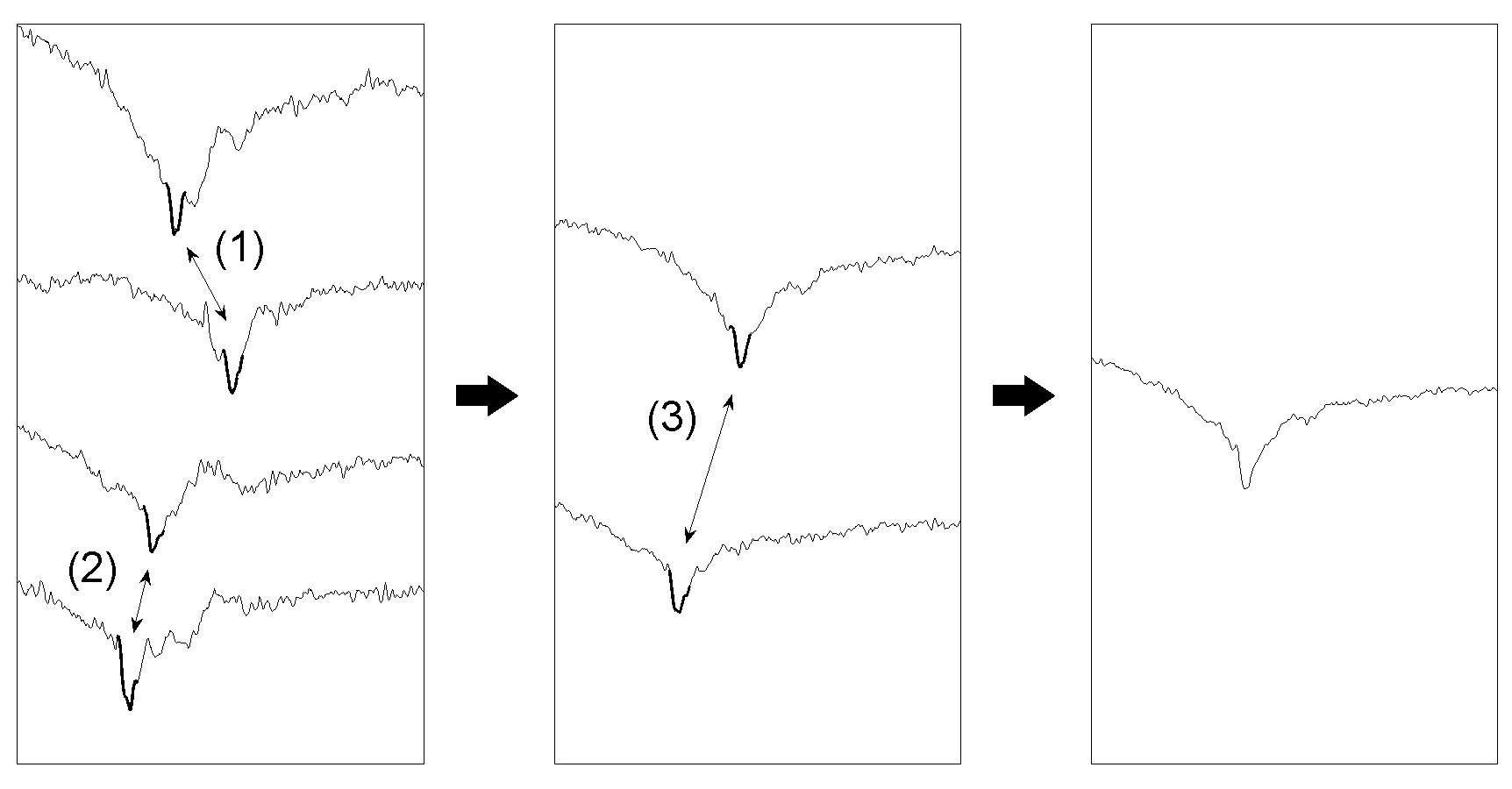

Supplement: S1 Fig — For both TS and ES, the 4 cortex traces with selected spikes were averaged using this procedure. The numbers correspond to the steps described in the text. In each step, two traces were shifted to overlap the most highly correlated signals (bold lines) and then averaged. (TIF) [file pone.0117289.s001.tif]

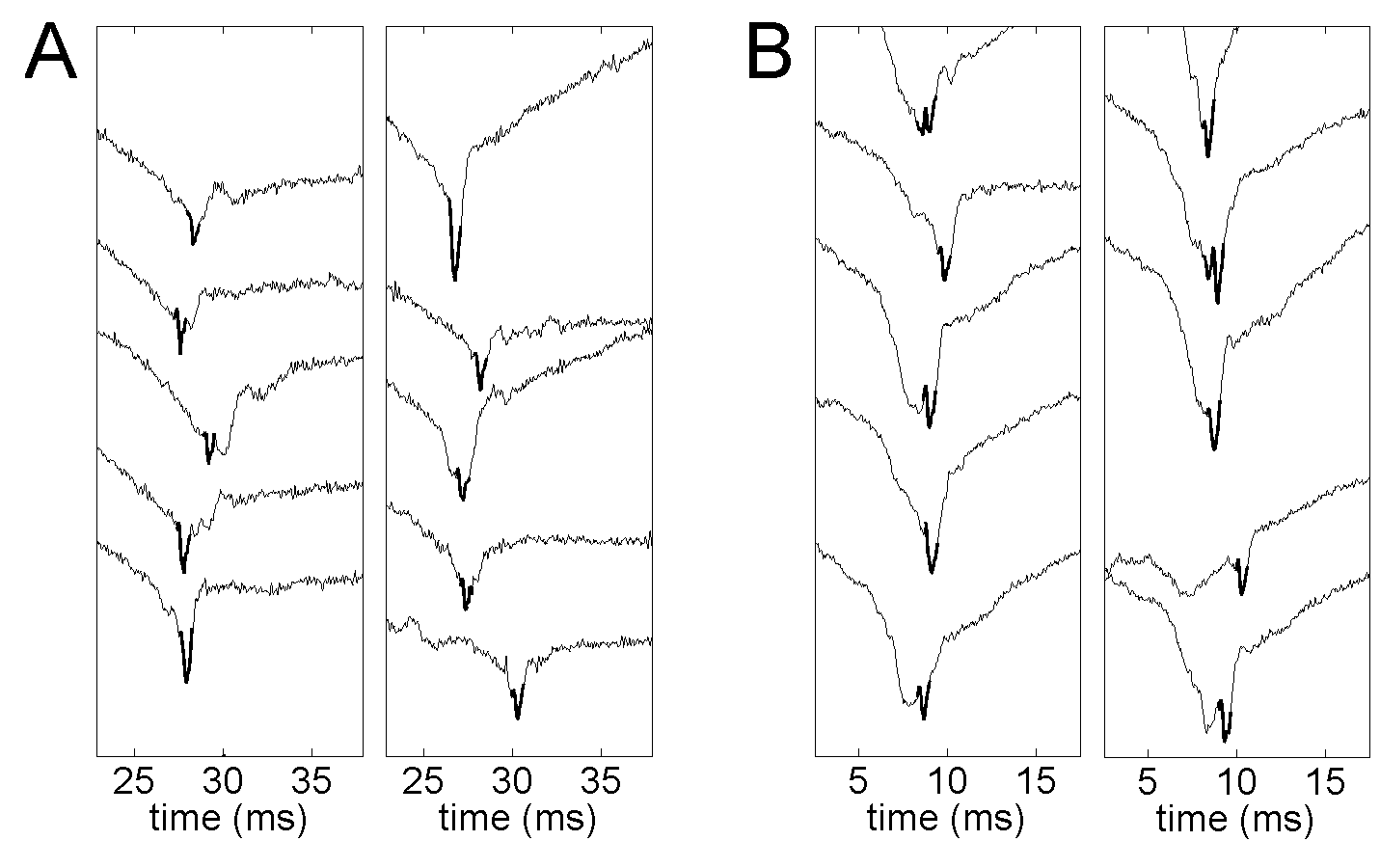

Supplement: S2 Fig — (A) TS-evoked spikes. (B) ES-evoked spikes. Bold lines show detected signals. (TIF) [file pone.0117289.s002.tif]
